# Successful Chimeric Antigen Receptor (CAR) T-Cell Treatment in Aggressive Lymphoma Despite Coronavirus Disease 2019 (CoVID-19) and Prolonged Severe Acute Respiratory Syndrome Coronavirus 2 (SARS-CoV-2) Replication - Case Report

**DOI:** 10.3389/fonc.2021.706431

**Published:** 2021-07-14

**Authors:** Verena Nilius-Eliliwi, Thomas Mika, Alexander Baraniskin, Max Wünnenberg, Marina Maslova, Christian Boy, Susanne Klein-Scory, Roland Schroers, Deepak Vangala

**Affiliations:** ^1^ Department of Medicine, Hematology and Oncology, Knappschaftskrankenhaus, Ruhr-University Bochum, Bochum, Germany; ^2^ Department of Hematology and Oncology, Evangelisches Krankenhaus, Hamm, Germany; ^3^ Department of Radiology, Neuroradiology, and Nuclear Medicine, Ruhr-University Bochum, Bochum, Germany

**Keywords:** SARS-CoV-2, CoVID-19, chimeric antigen receptor T-cells, diffuse large B-cell lymphoma, pneumonia

## Abstract

In patients with compromised immune function, severe acute respiratory syndrome coronavirus 2 (SARS-CoV-2) infection and coronavirus disease 2019 (CoVID-19) impose particular challenges. Especially in hematological malignancies, including lymphoma, the demands by this novel virus disease are further enhanced during sophisticated treatments, such as chimeric antigen receptor (CAR) T-cell therapy. Here, we present the first case of a patient with refractory diffuse-large B-cell lymphoma, who underwent CAR T-cell treatment in the context of SARS-CoV-2. Irrespective of prolonged and active SARS-CoV-2 infection, T cells were successfully isolated by apheresis and processed to anti-CD19 CAR T cells (axicabtagene-ciloleucel). In light of the aggressive lymphoma course, lymphodepleting chemotherapy and CAR-T cells were administered in early recovery after oxygen-dependent CoVID-19 pneumonia. Except for moderate cytokine release, this cellular immunotherapy was well tolerated. Notably, there is no deterioration of the SARS-CoV-2 infection; however, complete lymphoma response and full clinical recovery were observed. In conclusion, CAR T-cell treatment in aggressive lymphoma in the setting of SARS-CoV-2 infection is feasible and may offer significant therapeutic activity in refractory disease.

## Introduction

Despite rapidly increasing knowledge about severe acute respiratory syndrome coronavirus 2 (SARS-CoV-2) and coronavirus disease 2019 (CoVID-19), the ongoing pandemic remains a major challenge to health care workers around the world. Hematological patients represent a special subgroup who are prone for infections due to immunosuppression either by their underlying disease or intensive systemic treatment. Thus, management of these patients during the current pandemic can be challenging, especially if sophisticated treatment options, such as hematopoietic stem cell transplantation (1) and chimeric antigen receptor (CAR) T-cell therapy (2) are considered as only curative options.

Up to now, only two cases of SARS-CoV-2 infection in the context of CAR T-cell therapy have been reported in the literature. In one patient suffering from multiple myeloma, fatal outcome after BCMA-directed CAR T-cell therapy due to prolonged SARS-CoV-2 RNAemia and acute respiratory distress syndrome (ARDS) has been described (3). The other reported patient received the same anti-BCMA CAR T-cell therapy for multiple myeloma and developed a sustained antibody response and survived SARS-CoV-2 infection (4).

Patients treated with CAR T-cells are immunocompromised because of several lines of prior antineoplastic treatments including monoclonal antibodies. Moreover, lymphodepleting chemotherapy is generally administered before CAR T-cell transfusion, and CAR T-cells targeting lymphoma and myeloma deplete B-lymphocytes required for antibody production against SARS-CoV-2. Accordingly, patients suffering from B-cell neoplasia are particularly vulnerable to threatening infections such as SARS-CoV-2 (5, 6). On the other hand, delay of curative treatments, including immune cell therapy due to SARS-CoV-2 infection, exposes patients to the risk of progressive neoplastic disease and a fatal outcome.

In CoVID-19, the development of acute respiratory distress syndrome (ARDS) is linked to an overwhelming immune response, including cytokine storm, which founds the rationale for immunosuppressive therapies such as glucocorticoids and tocilizumab (7, 8). This might be the reason why some immunocompromised patients do not have a higher risk for severe CoVID-19 pneumonia as compared with healthy individuals (9). Of notice, for most CAR constructs, excessive cytokine release syndrome (CRS) remains also a serious complication. Interestingly, CRS following CAR-T cell therapy shows striking similarities to systemic inflammation response syndrome (SIRS) in SARS-CoV-2 infection (10, 11). SIRS in the context of SARS-CoV-2 infection cannot be easily distinguished from hyperinflammation subsequent to CAR T-cell therapy. Accordingly, management of CAR T-cell therapy in the context of SARS-CoV-2 poses a novel challenge.

Here, we present, to the best of our knowledge, the first patient with diffuse large B-cell lymphoma, who underwent CAR T-cell treatment with axicabtagene ciloleucel (axi-cel) in the context of SARS-CoV-2 infection. Prolonged SARS-CoV-2 replication and serious CoVID-19 pneumonia forced discontinuation of cytotoxic chemotherapy resulting in threatening lymphoma progression. Despite high tumor burden before axi-cel therapy, the patient developed complete lymphoma remission and experienced full clinical recovery. In this report, we describe the clinical course, including follow-up of SARS-CoV-2 respiratory load and demonstration of CD19-CAR T-cell expansion in peripheral blood. The abovementioned aspects of CAR T-cell immunotherapy in patients with B-cell lymphoma and SARS-CoV-2 are discussed.

## Case Description

**Figure 1:**
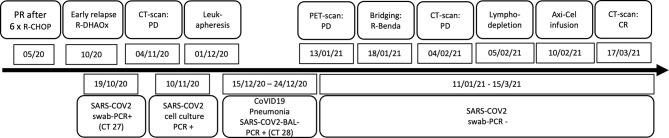
Timeline of diagnosis and treatment.

A 55-year-old patient was diagnosed with diffuse large B-cell lymphoma (DLBCL, GCB type) in December 2019 with affected lymph nodes in the mediastinum and abdomen (Ann-Arbor IIIA, IPI score 5, high-intermediate risk). He had been working as coal miner for more than 20 years, and he suffered from coronary artery disease (PTCA/DES in RCX/M3), chronic heart failure (ejection fraction 35%, AICD *in situ*), diabetes mellitus, arterial hypertension, thromboembolic disease (pulmonary embolism), and chronic obstructive lung disease (GOLD II). Following initial immunochemotherapy with R-CHOP (six courses, until May 2020) partial response (PR) was documented. Five months later, lymphoma progression with retroperitoneal, abdominal, inguinal, and liver (segment II) involvements was detected by computed tomography (CT) scans. Aiming for high-dose chemotherapy and autologous hematopoietic stem cell transplantation, one course of salvage chemotherapy comprising rituximab, dexamethasone, high-dose cytarabine, and oxaliplatin (R-DHAOx) was administered at the beginning of October 2020. A few days later (October 19), SARS-CoV-2 was detected in a PCR test of a routine nasal/pharyngeal swab sample (**Figure 1**).

Following standard operating procedures at our hospital, the patient was isolated and closely monitored. However, at this point, he did not develop any symptoms of CoVID-19 (SpO_2_ stably >95% without oxygen therapy). Considering ongoing SARS-CoV-2 infection, the second course of chemotherapy was held back. Abdomen CT scan for staging was performed at the beginning of November showing progression of the intraabdominal lymphoma manifestations. At that time, the option of CAR T-cell therapy for DLBCL refractory to second-line chemoimmunotherapy was discussed. However, the patient developed intermittent episodes of fever (38.5°C maximum) and still had SARS-CoV-2 replicates measurable in nasal/pharyngeal swab samples repeated every 3 to 4 days (CT values, 27–30; **Figure 1**). Hence, leukapheresis for T-cell collection in preparation of CAR T-cell manufacturing was postponed, although SARS-CoV-2 RNA was not detectable in peripheral blood.

At that time, CT scans showed mild ground-glass opacities in both lungs. To further investigate SARS-CoV-2 infection, cells were harvested from nasal/pharyngeal swab and cultured for 3 days. In subsequent PCR assays, SARS-CoV-2 could be specifically detected by PCR, indicating ongoing active infection.

Considering the worsening clinical situation and rising LDH serum concentrations as signs of progressive DLBCL, leukapheresis for CAR T-cell production was ultimately planned regardless of SARS-CoV-2 persistence as determined in PCR tests from nasal/oropharyngeal samples every 3 to 4 days (CT- values, 28-30; **Figure 1**). On December 1, the patient underwent successful leukapheresis and was subsequently discharged without respiratory impairment but with intermittent fever, which was interpreted as lymphoma caused B-symptom. At the time of leukapheresis, a concentration of 0.3 × 10^9^/L lymphocytes was measured in peripheral blood. In total, 2.2 × 10^9^/L lymphocytes could be collected during leukapheresis.

**Figure 2:**
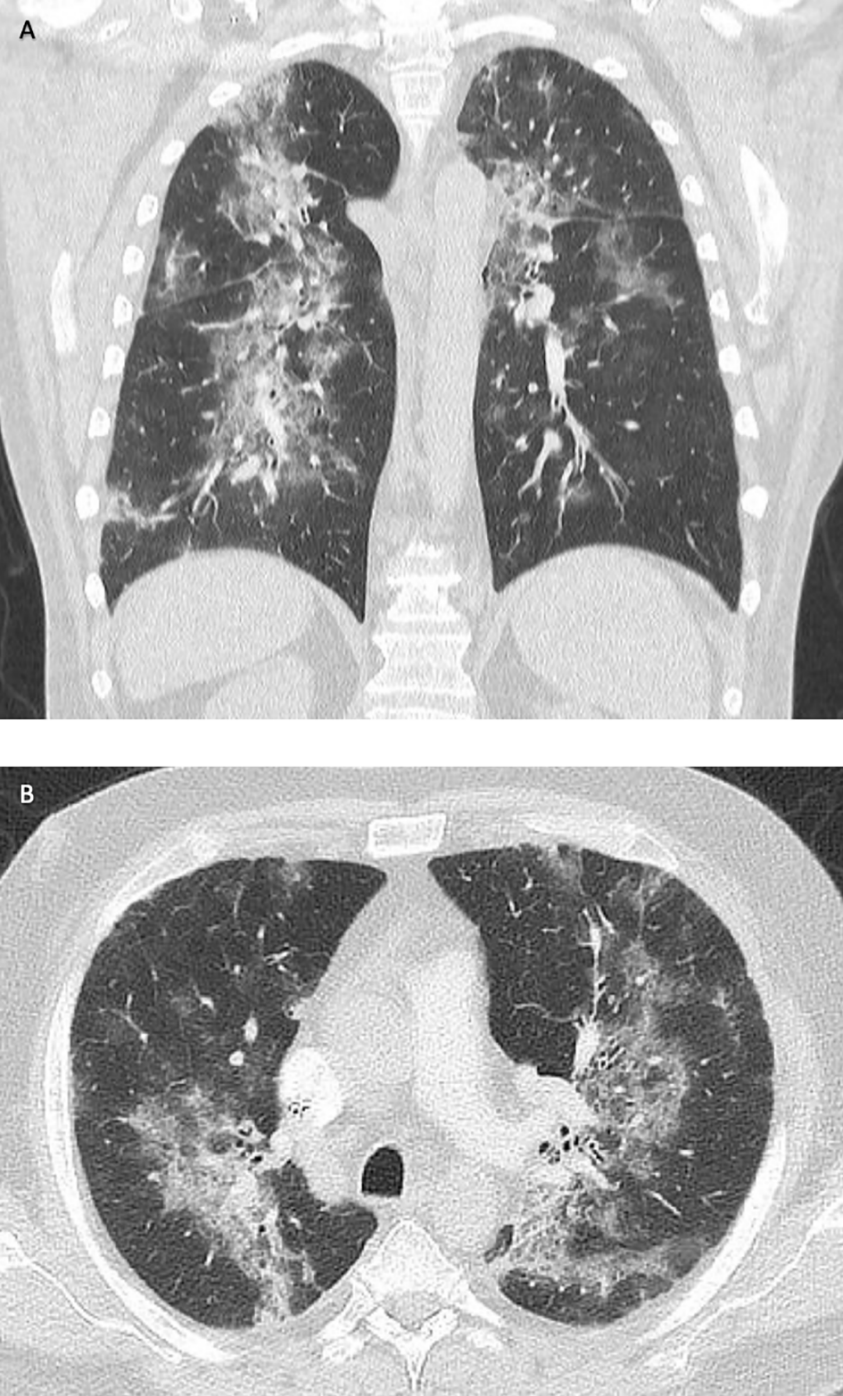
Computed tomography (CT) of the lungs in frontal **(A)** and transverse sections **(B)**. Disseminated ground-glass opacities due to COVID-19 pneumonia (December 2020).

Ten days later, the patient had to be readmitted because of increasing dyspnea. At that time, he required oxygen insufflation without mechanical ventilation. Chest CT scan demonstrated immensely progressive ground-glass opacities (**Figure 2**). Although SARS-CoV-2 PCR assays from nasal/pharyngeal swabs were negative, SARS-CoV-2 could be detected in bronchoalveolar lavage (BAL) samples at high concentrations (CT value, 28 in BAL; **Figure 1**). The patient received intensified treatment including dexamethasone and antibiotics. Two weeks later, he was discharged with continuous oxygen insufflation (2–4 l O_2_/min). Spirometry and bodyplethysmography indicated combined obstructive and restrictive ventilation disorder with decreased Tiffenau-index (62%) and FEV1 (47%), respectively. Vital capacity was reduced to 67% of the reference.

The SARS-CoV-2 PCR tests from nasal/pharyngeal swab stayed negative but so did also the repeated antibody tests (ACOV2 chemoluminescence, ROCHE). Six weeks later, the CAR T-cell product was ready for application.

**Figure 3:**
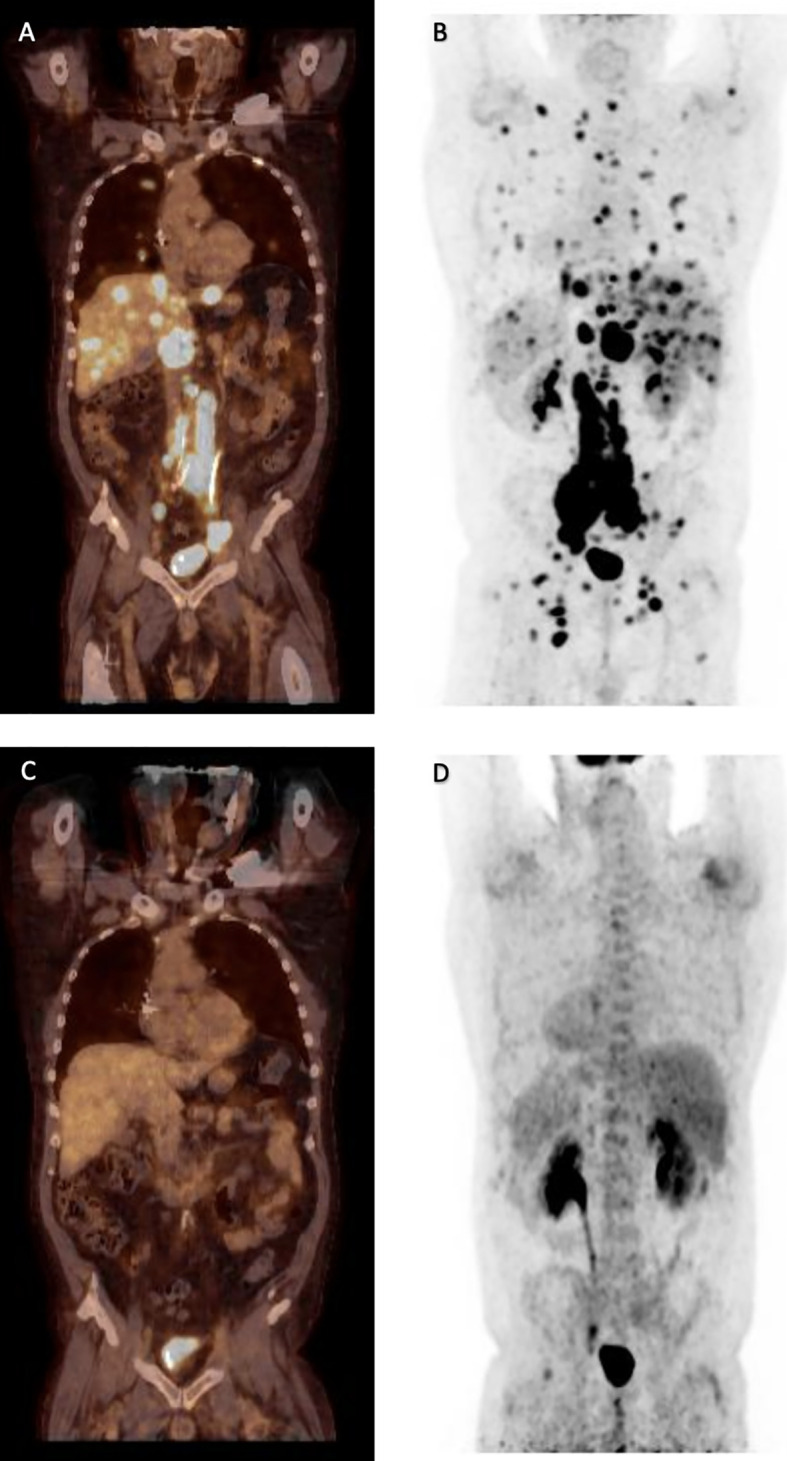
Positron emission tomography with **(A, C)** and without computed tomography co-registration **(B, D)** in ventral **(A, C)** and dorsal view **(B, D)**. Disseminated manifestations of diffuse large B-cell lymphoma refractory to chemoimmuntherapiy prior to CAR-T cell therapy **(A, B)** and in remission three months after anti-CD19 CAR-T treatment with axi-cel.

**Figure 4:**
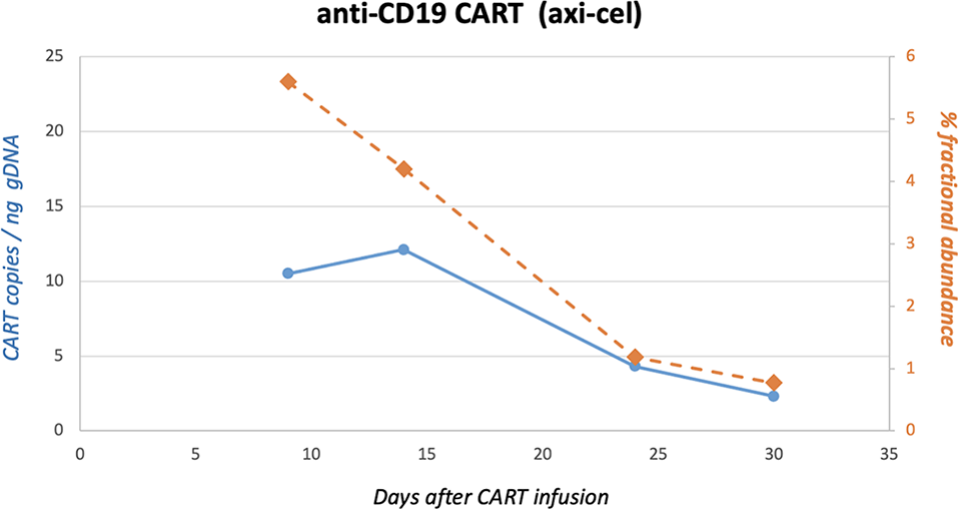
Quantification of CD19-CAR gene sequences in peripheral blood by ddPCR. CD19-CAR (axi-cel) gene sequences were quantified by digital-droplet (dd) PCR. In brief, genomic DNA was extracted from peripheral blood mononuclear cells at different time points after infusion of axi-cel. DdPCR was carried out to detect FMC63-28Z-1 amplicons as recently published by our group (13).

At this time, the patient suffered from new edema of both legs and increasing LDH concentrations (maximum LDH 345 U/l). A PET-CT scan performed with (^18^)F-labelled fluoro-deoxy-d-glucose (FDG) prior to CAR T-cell treatment demonstrated a massive progression of DLBCL with disseminated lymphoma manifestations and explaining the clinical symptoms (**Figures 3A, B** **)**. Indeed, bridging cytotoxic treatment with rituximab and bendamustin had shown no positive effect on lymphoma growth. Therefore, lymphodepleting chemotherapy with fludarabine (90 mg/m^2^) and cyclophosphamide (1500 mg/m^2^) was administered, and on February 10, CAR T-cells directed against CD19 (axicabtagene-ciloleucel; Yescarta®) were infused. Treatment was carried out on a special hematological ward, including respiratory and cardiovascular monitoring. During lymphodepleting chemotherapy and after CAR-T treatment, SARS-CoV2 tests from nasal/pharyngeal swabs were repeated twice weekly and remained negative. Subsequently, the patient developed CRS with maximum grade 2 (temperature, 38.5°C; mild hypoxemia, tremor) (12). CRS persisted for approximately 72 h, and tocilizumab had to be given twice (day +9). SARS-CoV-2 infection or reactivation, respectively, were excluded by regular PCR tests from nasal/pharyngeal swabs. On day +14 after CAR T-cell therapy, the patient was discharged in a good general condition without peripheral edema. Whole-body PET-CT scan 1 and 3 months later (days +30 and +90) showed a complete response (CR) with only residual lymph nodes without metabolic activation (**Figures 3C, D** **)**. The side effects and clinical response were in agreement with the expansion of anti-CD19 CAR T-cells in quantifications by ddPCR, as has recently been reported by our group (**Figure 4**) (13).

## Discussion

The SARS-CoV-2 pandemic raises several questions, which physicians these days have to answer without substantial supporting evidence. Treating relapsed/refractory (r/r) DLBCL imposes a therapeutic challenge even without an ongoing pandemic for hematologists around the world. Although longtime remissions can be achieved for more than 60% of patients after initial diagnosis, cure is less likely in r/r disease. Salvage immunochemotherapy, high-dose chemotherapy followed by autologous stem cell transplantation or allogeneic transplantation are potential treatment options. The long-term outcome for all these therapies has been unsatisfying either due to the high risk of another relapse or substantial non-relapse mortality in the case of allogeneic stem cell transplantation (14). Fortunately, the recent approval of CAR-T cell products has improved prognosis for patients suffering from r/r DLBCL with a satisfactory side effect profile.

Patients with lymphomas are prone to infections, on the one hand, because of the immunodeficiency caused not only by their disease but also by any systemic treatment leading to immunosuppression. Different analyses have shown higher morbidity and mortality rates for CoVID-19 patients suffering from underlying hematological malignancies compared with the general population, especially if active or refractory diseases is present (15–17). Furthermore, the length of in-hospital stays for hematological patients suffering from CoVID-19 is prolonged (5, 18).

In the context of SARS-CoV2, there are two major implications. First, SARS-CoV-2 infection must be prevented in patients undergoing intensive immunosuppressive therapies by all means. In our institution, a strict protocol has been established, which includes structured history taking regarding CoVID-19-symptoms and contact to infectious peers, as well as PCR testing before admission. Fortunately, by this, for the vast majority of our patients, SARS-CoV2-infections have been prevented, with our here presented patient being one of the very few exceptions. Second, treating physicians have to decide on when to initiate or continue treatment for a potentially life-threatening disease in case of SARS-CoV2-infection. Because of the novelty of CAR T-cell therapy, there are no data to base this decision on. To date, only two cases of CAR-T cell treatment have been reported in the context of SARS-CoV2-infections, both in myeloma patients and both with different outcome (3, 4).

The case presented here is, to the best of our knowledge, the first r/r DLBCL undergoing CAR T- cell therapy in the context of CoVID-19. As has been shown before, viral replication in our patient was persistent for a longer period than that observed in immunocompetent patients (19–21). Respiratory symptoms only developed about 56 days after the first detection of SARS-CoV-2. The intermittent fever might have been a sign of CoVID-19 but more likely was a B-symptom associated to lymphoma progressing in spite of salvage immune-chemotherapy.

Risks and benefits for conducting intensive therapeutic measures, such as CAR-T-cell treatment in this clinical setting, have to be carefully weighed. Reports differ regarding outcome of SARS-CoV-2 positive lymphoma patients receiving B-cell–depleting treatments, such as rituximab (18, 19). We had different reasons for conducting leukapheresis: our patient showed disease progression despite intensive and usually effective salvage therapy, thus qualifying for CAR T-cell therapy. On the other hand, patient’s cardiac and pulmonary history were important aspects not to pursue other intensive treatment options, such as allogeneic stem cell transplantation. Although viral replication could be detected at the time point of apheresis, CT values had been increasing over time, and the patient had hardly developed any symptoms. Furthermore, RNAemia was ruled out.

However, severe respiratory symptoms developed 2 weeks after apheresis. Neither monoclonal antibodies nor convalescent plasma or remdesivir were administered, as the patient stabilized quickly. Furthermore, the patient had antibody deficiency syndrome and did not develop detectable antibody titer against SARS-CoV-2. Fortunately, pneumonia resolved, and virus replication seized. As the patient’s lymphoma was rapidly progressive and refractory to chemotherapy at that point in time, we decided to initiate lymphodepleting chemotherapy and infuse the CAR-T-cells. The patient developed grade II CRS, which was successfully treated with tocilizumab. Re-activation of SARS-CoV-2 did not occur.

This case highlights another important aspect of CAR T-cell therapy: as for other consolidating treatments in hematological malignancies, recent data indicate that reduction of tumor load prior to CAR-T-cell treatment is associated with a better outcome and a lower rate of treatment specific adverse events, such as CRS and ICANS. Not only measurable tumor mass in imaging studies but also surrogate parameters, such as lactate dehydrogenase (LDH), have been associated with outcome (EBMT 2021). As these observations are probably true, there is obviously a subgroup of patients benefitting from this treatment despite high LDH and extensive lymphoma. This highlights the need for better biomarkers. Also, patients should not *per se* be excluded from CAR T-cell therapy if a high lymphoma burden or high LDH levels are present.

Our patient showed a complete remission only 35 days after CAR T-cell infusion. Early CR in turn has been associated with a high probability of long-term survival (22). CR was confirmed 3 months after CAR-T treatment. Compared to all previous treatments, this is the best response our patient ever had in his treatment history. A reason for this might be that the patient received only two lines of chemo-immunotherapy prior to apheresis and myeloablative regimens were not administered. This might have been beneficial for T-cell function (23).

In summary, we present the first case of r/r DLBCL successfully treated with CAR T-cell infusion despite SARS-CoV-2 infection, extensive comorbidity, and high lymphoma burden. Considering potential underreporting of unsuccessful clinical courses, the benefits and risks of CAR-T treatment in the context SARS-CoV2 certainly have to been addressed in future clinical studies.

## Data Availability Statement

The original contributions presented in the study are included in the article/supplementary material. Further inquiries can be directed to the corresponding authors.

## Ethics Statement

Written informed consent was obtained from the individual(s) for the publication of any potentially identifiable images or data included in this article.

## Author Contributions

VN-E, RS, AB, MW, MM, CB, and DV collected the clinical and laboratory data. All authors discussed the data. SK-S performed the ddPCR assays. MM and CB performed and interpreted the PET-/CT scans. VN-E, RS, and DV wrote the manuscript. All authors contributed to the article and approved the submitted version.

## Conflict of Interest

The authors declare that the research was conducted in the absence of any commercial or financial relationships that could be construed as a potential conflict of interest.
